# Evaluating GenAI‐produced feedback on undergraduate bioscience essays against good higher education feedback practice

**DOI:** 10.1002/2211-5463.70339

**Published:** 2026-09-04

**Authors:** Annabel Court, Nigel Francis, Andrew Shore, Stephen Rutherford

**Affiliations:** ^1^ School of BioSciences Cardiff University UK; ^2^ School of BioSciences, Faculty of Science The University of Melbourne Australia

**Keywords:** artificial intelligence, assessment, chatgpt, feedback, GenAI, Generative AI

## Abstract

The emergence of generative artificial intelligence (GenAI) has led to substantial interest in its potential to support student learning and assessment in higher education. In particular, GenAI's ability to provide immediate and iterative formative feedback on the quality of students' work has substantial potential to enhance student learning and attainment. However, for feedback to be impactful and useful, the quality and utility of feedback generated by GenAI needs to accurately reflect the quality of the work and align with principles for good feedback provision. This study evaluated the feedback that an undergraduate student might obtain from a single, unscaffolded interaction with a GenAI platform (chatgpt4o), without tutor input, applied to 30 Year 1 Bioscience essays. Using a bespoke rubric for feedback quality alongside qualitative and quantitative analyses, the GenAI‐generated feedback was evaluated for alignment to established hallmarks of good feedback practice. Overall, GenAI‐authored feedback had useful elements but was limited in scope. While feedback tended to be accurate, specific to the essay, with a strong focus on content and substantial elements of feed‐forward advice, critical guidance and motivational feedback were limited. Within the feedback test, neutral observations focused mostly on structure and use of figures. Positive feedback focused mainly on use of language, and areas for improvement mostly on structure, missing content, and citations/references. These findings suggest that GenAI may have some potential to provide instant, formative guidance to students, but its use may require substantial scaffolding by educators in order to be effective.

## Graphical abstract

This pilot study investigates the potential of Generative AI to provide formative feedback to students. chatgpt was prompted to provide feedback on Year 1 Bioscience essays, which were evaluated against established good feedback practices. GenAI‐authored feedback had useful elements, but was limited in scope. GenAI may have potential to provide instant, formative guidance to students, but requires scaffolding by educators.

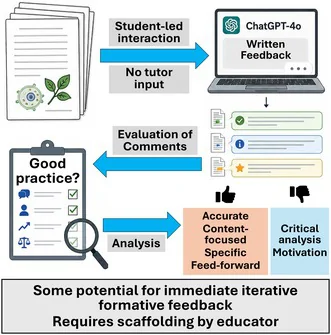

**Abbreviations**
GenAI: generative artificial intelligence
HE: Higher Education
LLM: large language model
SD: standard deviation

The emergence of Generative Artificial Intelligence (GenAI) has had a profound impact on Higher Education (HE). There has been considerable discussion regarding the potential advantages and threats of GenAI ‘Large Language Models’ (LLMs) regarding learning and the assessment of academic standards. A key focus of the current literature is the risk to academic integrity and the potential overreliance on these tools [1, 2, 3]. However, GenAI also offers significant opportunities for assessment and learning. The pattern‐recognition and comparative abilities of GenAI platforms suggest a potential for these platforms to be used to evaluate written academic work, compared against expected disciplinary norms. While research is currently investigating whether GenAI can replicate human perceptions of quality when assessing student outputs, its ability to identify more subtle omissions, or suggest areas for potential improvement in lengthy, technical scientific writing, remains the subject of debate [4, 5, 6].

In an increasingly pressurised educational environment within HE, opportunities for students to receive formative feedback and support from academic staff are often limited. This paucity of support can have a detrimental impact on the development of academic skills by students. The sector‐wide issues around the provision of feedback by educators [7], and its reception and use by learners [8, 9, 10], are evidence of the challenges the sector faces from limited educator‐learner interactions. When adopted effectively, the feedback process can highlight progress and attainment to students and illustrate what strategies they need to adopt to improve [11]. Feedback literacy requires the ability of the learner to self‐reflect on their performance, but in order to do this, an accurate and accessible evaluation of their performance is required [12, 13].

There is a strong argument that feedback is most impactful when provided at a point where the student can use the guidance to improve their work prior to submission of the assignment [14]. Such ‘formative’ feedback, prior to submission of the assignment, should ideally involve interaction between the learner and educator [15]. This interaction should ideally be interactive, dialogic and provide the basis for iterative improvements on the work [16]. Feedback should therefore provide a scaffold for improvements and iterative reflection on the process. However, in practice, workload pressures and student numbers often prohibit this kind of iterative interpersonal interaction for formative feedback in most assignments [17, 18] and typically prohibit multiple cycles of feedback, reflection, revision and resubmission for more feedback. There is a potential that GenAI could be used to replace the educator in that iterative feedback loop at the formative stage and provide guidance iteratively as the student progresses through an assignment. Without the educator workload implications of repeatedly reviewing and providing feedback on iterations of an assignment, GenAI can instead be re‐prompted as many times as the student wishes, potentially providing numerous iterative revision cycles. GenAI therefore has the potential to empower students to gain extensive guidance on their work, thus self‐evaluating their performance more effectively prior to submission of an assessed piece of work, without the input of time‐poor academics [19].

In order for GenAI‐produced formative feedback to be used iteratively by the student, the feedback guidance provided needs to be of sufficient detail, accuracy and depth to support improvements to the work. The purpose of this project was to investigate the potential use of GenAI by an undergraduate Bioscience student for obtaining formative feedback, without the ‘human‐in‐the‐loop’ involvement of the educator. The project aimed to evaluate the quality of GenAI‐generated feedback on written coursework, benchmarking it against generally accepted best feedback practices within HE. This study was not concerned with how an educator might coproduce feedback using GenAI, nor with how a student might subsequently receive and act upon such feedback, but rather the more basic question of what output a student might obtain from a single, unscaffolded interaction with an LLM, without any tutor input. Although the assumption is that a student might use sequential iterations of GenAI guidance, followed by revisions to the manuscript, followed by a repeated generation of GenAI feedback on the revision, this study was focused on a single point of interaction with GenAI. The study aimed to provide an indicative snapshot within an iterative feedback process. The project had two objectives: firstly, to identify whether GenAI was capable of providing useful feedback that evidenced hallmarks of good feedback practice [20, 21]; secondly, to identify patterns or systemic biases within the GenAI‐generated feedback.

Feedback was generated on a sample of bioscience essays using openai's chatgpt4o. Using a rubric developed to align with contemporary models of good feedback practice, the quality of the GenAI‐generated feedback artefact was evaluated. It was observed that feedback provided in the GenAI output was in‐keeping with good feedback practice in some aspects (highlighting areas of strength and weakness, and advising future actions for improvement). However, there was a strong emphasis on structural comments relating to content and format, with more‐limited scope regarding the identification of higher‐order critical skills. The extent to which the GenAI feedback included motivating comments for the student to improve was also limited, as was feedback relating to analysis and creativity. These findings suggest that GenAI could be used formatively by students to improve their writing styles and presentation, but would be limited in supporting the higher‐order skills that are the hallmarks of good academic writing.

## Methods

### Essay subjects, choice of GenAI platform and ethical approval

The essays used in this study were from a pool of submissions for a Year 1 (Level 4) Bioscience written assignment at a UK university. Each essay addressed one of 21 potential questions with a 1500‐word limit. Essays were submitted to the university's virtual learning environment in the spring of 2024.

Student authors provided consent, via a robust opt‐out process, for their essay to be used for research, and entered into chatgpt4o. 450 out of 482 students provided consent. The project received ethical approval from the Cardiff University School of Biosciences Research Ethics Committee (Reference: Francis 24‐08‐03). All essays were anonymised prior to use in the research.

chatgpt4o (accessed through subscription) was used as the LLM for the study, as chatgpt4o was the most widely used LLM among UK students at the time of the study. Default model settings (including temperature) were used, and the provider's memory feature was disabled.

### Iterative prompt engineering

The development of the prompt for generating effective feedback from the LLM was an iterative approach, using a single first‐year biology essay. The initial prompt was simple and was repeated three times to assess the consistency of the generated outputs. Specific descriptors and contextual elements were then introduced to align the feedback process more effectively to the context of a Year 1 undergraduate essay. Variations included specifying the academic level, subject and evaluator perspectives. The prompts explicitly requested feedback to be structured around knowledge, comprehension, application, analysis, synthesis and evaluation and were further refined to specify a ‘persona’ for the LLM, emphasising its objectives and tone, such as acting as a prompt enhancer and a GenAI writing tutor. The final prompt version (‘Prompt E’) included a request that the GenAI quote sections of the essay where needed and provide revised examples. Prompts adopted at each stage are summarised in Data S1.

### Development of a rubric for evaluating GenAI feedback

A rubric was created to evaluate the quality of GenAI‐generated feedback, aligned with best practices in contemporary academic feedback literature in Higher Education. The rubric was developed in four phases: establishing a theoretical framework, designing the initial rubric criteria, pilot testing, refinement and validation.

To establish the theoretical framework, a small‐scale scoping review of the feedback literature was undertaken using the Education Resources Information Centre (ERIC) and Scopus databases. Six criteria were identified from this review (Table 1), as relevant to assessing GenAI‐generated feedback. The criteria highlighted feedback related to practical competences, strategies and fostering student autonomy and long‐term self‐regulation. For the purpose of this rubric, motivational feedback was defined as language intended to support the affective and self‐regulatory dimensions of learning. This encompassed encouragement, affirmation of effort and progress, and framing that supports self‐efficacy and growth orientation [22, 23, 24].

**Table 1:** Criteria for evaluating feedback and their justifications. A table outlining six key criteria identified in the literature for evaluating feedback quality, along with their respective justifications.

Criterion	Justification
Accuracy of feedback	Verifies whether the feedback is factually correct and aligns with the content of the essay [47]
Focus on content	Identifies how the content contributes to the essay's overall success. Effective feedback should be explicit and example‐driven [48]
Specificity, clarity, and detail	Highlights that responses need to be precise and meaningful to the student. This reflects the principle of learner centred education [13]. It also reduces ambiguity in feedback, caused by the disconnect between student interpretations and instructor intent [12]
Constructive and critical	Prioritises actionable feedback rather than surface‐level corrections, addressing concerns on the limitations of overcorrection [49]
Feed forward	Provides clear next steps for the student. It is consistent with a feedback model which identified that students found task, process and self‐regulation level feedback most helpful [31, 50]
Motivation	Motivation encourages the development of positive motivational beliefs and self‐esteem, which in turn increases participation [20, 23]. Research suggests that students' motivational frameworks shape their responses to feedback and their ability to self‐regulate learning [22]

The rubric was initially developed as a 4‐point scale and tested using a sample of AI‐generated feedback responses from the chatgpt4o model using a sample of first‐year biology undergraduate essays answering either of two questions (5 essays per question). This initial pilot suggested that a 4‐point scale was too limited to be discerning, so Level 5 (‘Exemplary’) descriptors were added to create a 5‐point scale:

- Level 1: below expectations
- Level 2: approaching expectations
- Level 3: meets expectations
- Level 4: exceeds expectations
- Level 5: exemplary

Descriptive examples were added to illustrate what feedback at each level might look like (see Table S1 for the rubric).

Piloting of the 5‐point rubric was repeated using the same 10 essays, with two researchers independently applying the rubric to the 10 feedback outputs generated by GenAI. The essay titles were either a broad discursive topic essay on the role of children in the spread of COVID‐19, or a more fact‐based question regarding neuroscience‐focused content. To generate pilot feedback, each essay was fed into chatgpt4o with the final prompt version. This produced 10 separate feedback outputs in total. A comparison of the variance among the two sets of scores (Researcher 1 and Researcher 2) was conducted using Levene's test. The difference in mean scores between the broad and more specialised essay topics was also calculated.

### Application of the rubric for GenAI‐generated feedback

A larger set of essays (*n* = 30) was used to generate GenAI feedback using the same final prompt for chatgpt4o. This set of essays included the 10 samples used in the pilot, with the remainder selected from the remaining essays of the two subjects chosen for the pilot. Essays were selected to follow the mark distribution for the overall pool, as closely as possible, to provide a range of essays of varying qualities (see Fig. S1). The rubric was applied by Researcher 1 to each of these GenAI feedback outputs for these essays.

The final prompt developed from the prompt design process (‘Prompt E’) was used. Each essay was processed in a separate chat, so that outputs were independent. Feedback was generated with chatgpt4o on a paid subscription, using the default user interface and settings (e.g. temperature). Student essays were uploaded individually, as separate PDF attachments, and given the same prompt. No responses were discarded, regenerated or otherwise excluded. The memory feature was disabled, with conversations reset between essays (each essay was prompted in a new chat), so that information could not be carried across responses.

The prompting approach was designed to mimic, as closely as possible, the most likely approach taken by a typical undergraduate student. The marking rubric was not included in the upload to the LLM, in order to align the approach with the evidence from the literature that engagement with marking criteria by undergraduate students is typically minimalistic [25, 26, 27]. The LLM was therefore asked to provide feedback without reference to marking criteria.

A single output was generated for each essay. None of the responses generated were reprompted or excluded from the analysis. Again, this approach was adopted because it best reflected how a student might use GenAI in practice. Studies suggest that a student is most likely to engage with GenAI tools by using concise, single prompts with minimal iterative refinement and typically accepting the first response returned [28, 29]. The artefact evaluated here is, in most cases, representative of the feedback a student would likely receive.

For the expanded dataset, the mean, standard deviation and Pearson's correlation coefficients were calculated to examine the relationships between the quality determinations of the GenAI feedback against the rubric.

### Qualitative analysis of feedback composition

A thematic analysis [30] was conducted on eight feedback outputs, selected based on their representing a range of feedback quality scores. Codes describing feedback types were generated using nvivo 14. The codes were grouped into three categories: neutral/generic comments, positive comments/strengths and negative comments/suggested improvements. For each of these three categories, the percentage word coverage of each feedback comment type was calculated. As a proportion of the total word count for that category.

## Results

### Iterative prompt engineering enhanced feedback detail

Prompt A includes three sequential iterations of this very basic prompt created generalised feedback that covered strengths and weaknesses but lacked depth. Prompt A identified issues without providing solutions and did not indicate the quality of the work. Prompt A was therefore adjusted to encourage a more detailed response: ‘Prompt B’.

Prompt B included the addition of descriptors of the assignment, a context of the target audience, and context of the feedback provider (for the GenAI persona to mimic), ensured that the feedback included actionable critiques of the student work. The tone and expectations of the feedback was more suited to a Year 1 undergraduate, with emphasis on fundamental understanding, clarity and logical structure.

In order to cater to students with more advanced essay writing skills, the prompt was engineered to request feedback on different depths of knowledge where applicable (‘Prompt C’). However, the responses struggled to advance to higher levels of thinking (argument logic, interpretation of research and critical evaluation/synthesis).

Framing the chatbot's role as being instructional (‘Prompt D’) ensured that the feedback was more constructive and focused on learning, echoing human feedback phraseology. Prompt D provided responses that included suggestions for approaches to improve students' learning. Prompt D also balanced critique with encouragement.

The final prompt (‘Prompt E’) was chosen because it created actionable, structured responses that encouraged rhetorical analysis. It included a description of the role the GenAI should assume, and a request for precise feedback, creating detailed responses that quote the essay and giving examples that might make the feedback useful for revision. This iterative process ensured that the prompt could create the clearest and most comprehensive feedback, superior to the vague alternative.

### Piloting the rubric

**Fig. 1:**
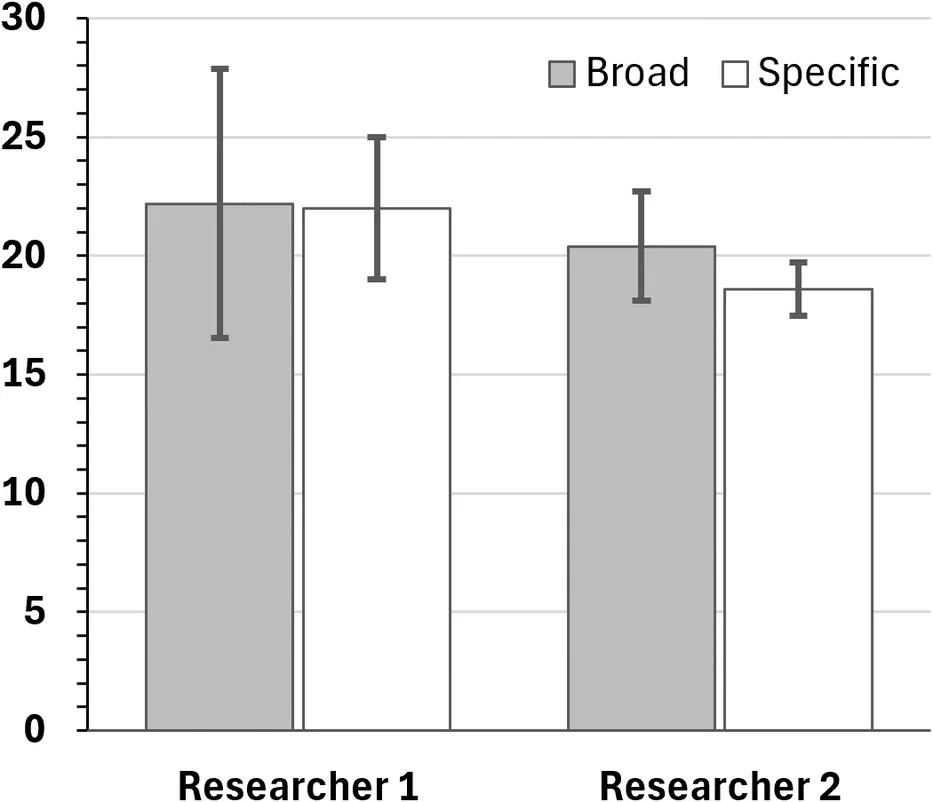
Total scores for GenAI‐generated feedback quality. Comparison of the total scores from 6 criteria (rated on a scale of 1–5) for 5 essays with a broad subject focus (grey bars) and 5 essays with a specific focus (white bars), assessed by two independent researchers. Data shown are the means of the 5 essays in each set. Error bars = standard deviation.

The rubric was designed to cover a 5‐point scale across the 6 criteria. The rubric was piloted for the ability to be discerning against feedback responses for 5 essays, each of two essay questions, one with a specific subject and the other a broader topic. Researcher 1 differed in the outcomes of the rubric compared to Researcher 2 (see Fig. 1).

To assess the variability in total scores (Table 2), Levene's test was conducted to compare the variances for Researchers 1 and 2 (18.32 and 3.83), respectively; test statistic of 30.00, *P* = 1.30 × 10^−7^, indicating that the scores from Researcher 1 exhibit significantly greater variability in total essay scores compared to Researcher 2. However, the variability between individual criterion scores was much less, with a variance of 1.07 (Researcher 1) and 0.50 (Researcher 2). Levene's test showed no significant difference between the researchers (test statistic 1.71 and a *P*‐value of 0.194), indicating no significant difference between the raters (*P* > 0.05). This suggests that the rubric was sufficiently discerning in a consistent manner.

**Table 2:** Summary statistics from the initial pilot sample. Results of the initial pilot sample, comparing total scores (combined score of 6 criteria, each rated 1–5 for 10 essays) given by two researchers. Data include the range of total scores (lowest and highest scores recorded), mean, median, variance of individual scores (rated 1–5; *n* = 60) for individual criteria and variance of total scores (*n* = 10).

Marker	Range	Mean	Median	Variance in criterion scores	Variance in total score
Researcher 1	14–28	22.1	21.5	1.07	18.32
Researcher 2	17–23	19.5	19.5	0.50	3.83

### Feedback is accurate, specific and constructive, but lacks motivational aspects

When the rubric was applied to the test set of 30 essays, a comparison of the mean scores for each criterion (Table 3) indicates that GenAI‐generated feedback was relatively good quality for the substantive elements of the essay, such as accuracy, specificity and clarity, content, as well as future guidance. However, a deficiency was identified in the critical and motivational aspects of the feedback.

**Table 3:** Comparison of the mean scores for feedback quality criteria. Data from feedback provided by GenAI to 30 Bioscience essays, evaluated using the bespoke rubric, detailed by criterion.

Criterion	Mean score	St dev
Accuracy of Feedback	4.3	0.68
Specificity, clarity and detail	3.9	1.10
Focus on content	3.8	0.79
Feed forward	3.7	0.68
Constructive and critical	3.3	1.06
Motivational	3.1	1.45

The accuracy of the feedback generated gained the highest rating (mean = 4.3, SD = 0.68), indicating that AI‐generated feedback aligns well with the content of the essays. Specificity, clarity and detail (mean = 3.9, SD = 1.10) also scored highly, but with substantial variation in scores, suggesting that AI‐generated responses provided detailed and precise evaluations, but this was inconsistent. Focus on content (mean = 3.8, SD = 0.79), and feed forward (mean = 3.7, SD = 0.68) also scored well, indicating both backwards and forwards‐looking comments were included. The least well‐scored were constructive and critical comments (mean = 3.3, SD = 1.06) and motivational comments (mean = 3.1, SD = 1.45), indicating that GenAI was relatively poor at the more motivational and analytical elements of feedback, and there was considerable variation in this provision.

Pearson's correlation coefficients were computed to further investigate the relationships between the quality of different aspects of feedback to determine if there were trends within the GenAI feedback outputs (Table 4). One of the most prominent findings was the strong relationship between the accuracy of feedback and focus on content (*r* = 0.751, *P =* 0.012). This suggests that when feedback is aligned to the essay contents, it tends to focus more on the substantive content of the work. Another relationship was the high correlation between constructive feedback and motivation (*r* = 0.774, *P =* 0.009). Although motivational aspects were less prevalent overall in the feedback (Table 3), this correlation suggests that whenever motivational language does appear, it often arrives within or alongside constructive comments. Specificity, clarity and detail showed strong associations with feed forward (*r* = 0.703, *P =* 0.023) and constructive and critical (*r* = 0.696, *P =* 0.025). This finding suggests that when feedback is specific and detailed, it also tends to give directions for improvement. Correlation was also noted between accuracy of the feedback and it being constructive and critical (*r =* 0.637, *P =* 0.048) and specific (*r =* 0.643, *P* = 0.045).

**Table 4:** Correlation between feedback criteria. Pearson correlation coefficients between different between the scores allocated to feedback criteria (across 30 essays).

Criterion	Accuracy of feedback	Focus on content	Specificity, clarity, and detail	Constructive and critical	Feed forward	Motivation
Accuracy of feedback	1	0.751*	0.643*	0.637*	0.220	0.307
Focus on content		1	0.486	0.213	0.292	−0.078
Specificity, clarity, and detail			1	0.696*	0.703*	0.425
Constructive and critical				1	0.451	0.774**
Feed forward					1	0.261
Motivation						1

* *P* < 0.05.

** *P* < 0.01.

Overall, these correlation patterns highlight the importance of striking a balance between the different criteria. High accuracy and specificity can enhance the focus on content; however, without explicitly incorporating motivational elements, the overall impact of the feedback may be diminished. Providing constructive guidance and motivational remarks can help ensure that the feedback process is not only instructive but also supportive.

### Qualitative analysis shows greater emphasis on structural factors than critical skills

Coding of the comments across the eight pieces of feedback, placed them within 9 codes (see Table 5), covering either structural issues (content, use of figures, essay structure, language use, use of evidence/referencing), exemplars (rewritten snapshots from the essay, exemplars extracted from the essay) or critical comments (critical analysis, suggestions for improvement). A quantitative analysis of the comments thus coded reveals strong preferences for feedback that focuses on structural aspects of the essay. Critical analysis and means of presenting data via visualisations tended to be less commonly represented.

**Table 5:** Categories of feedback areas of focus. Codes identified by the thematic analysis covering the feedback given within the 8 feedback samples analysed.

Category	Explanation
Content	Accuracy and coverage of the scientific content within the essay, compared against the requirements of the essay title
Structure of essay/argument	Overall structure of the written component of the essay, including the development/presentation of an argument or thesis
Language	Use of general and scientific language and terminology; conciseness of expression and general clarity
Visualisations and figures	Use of figures, diagrams, tables and charts to support or enhance the written content
Use of evidence; citation/reference use	Use of evidence to support the content or argument. Use of citations to provide reference to the literature. Reference and citation format
Critical analysis and depth	The depth of coverage of the content and critical analysis applied to the content, argument or evidence. Conclusions and discussion
Excerpts extracted from the essay	Use of excerpts copied from the text of the essay to highlight a point being made in the feedback
Rewritten examples for improvement	Extracts taken from the essay and rewritten by the GenAI platform to exemplify better or alternative approaches
Suggestions for improvement	Specific suggestions of how to improve, either related to an individual element or generic/holistic advice. Potential future considerations

**Fig. 2:**
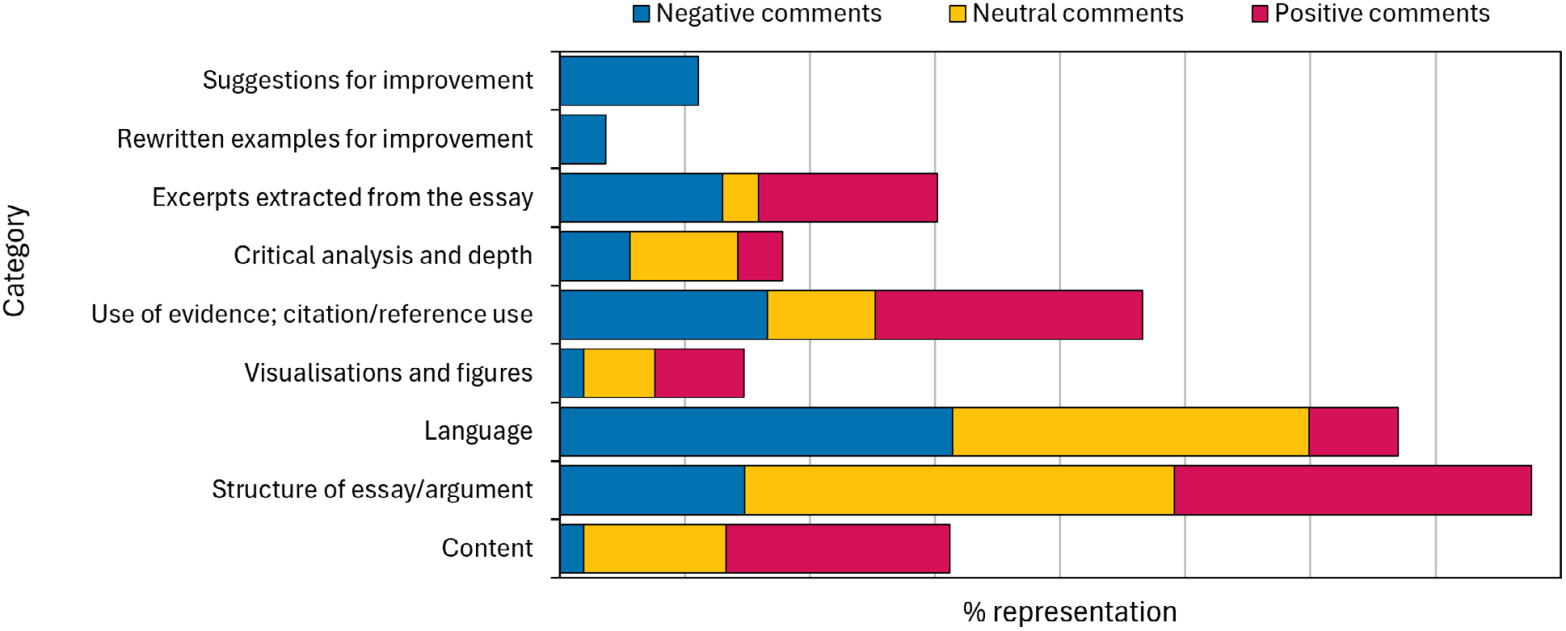
Distribution of areas of focus in the GenAI‐generated feedback. Relative distributions of the total wordcount for feedback aligned to feedback categories. Categories are divided between negative comments and critiques (blue), neutral comments and observations (yellow) and positive comments and encouragements (magenta). The vertical bars each represent 10% boundaries.

The text of the GenAI‐generated feedback was quantified based on proportionate coverage within the three categories (Fig. 2): (A) neutral/nonjudgemental observations and general comments, (B) positives and highlighted strengths and (C) limitations and specific suggestions for improvement. Most general observations focused on essay structure and the use of language within the writing, highlighting that the GenAI‐generated feedback tended toward the structural aspects of the work. When focusing on highlighting strengths within the essays, the feedback focused once again on essay structure primarily, but also tended to emphasise content and use of evidence and referencing, frequently including exemplars from the essay to reinforce those points. In contrast to the neutral comments, there was less focus on language within the highlighted strengths (possibly a reflection of the quality of language in the essays, however). There was still relatively little focus on critical analysis or depth, or visualisations. Suggestions for improvement once more focused on structure, evidence and language mostly, but with more emphasis on language than structure and evidence use. Very specific suggestions for improvement were substantial within this category, while being absent from the previous two, indicating that suggestions for improvement were limited to addressing deficiencies rather than further‐improving strengths. In some cases, specific extracts from the essays were used to exemplify how to improve. What was conspicuous, by their relative absence, in the areas for improvement were comments relating to missing content. This relative absence either indicates that the essays themselves were comprehensive in their content (which would not align with the marks the essays received from this criterion) or that the GenAI was not able to clearly identify omissions.

## Discussion

The findings of this study suggest that while GenAI‐generated feedback currently lacks the comprehensive depth of human academic appraisal, it offers a functional, albeit limited tool for supporting formative assessments in the biosciences. The usefulness of GenAI is primarily confined to the accuracy of content and structural elements of writing, such as language use and referencing. However, its inability to evaluate higher‐order thinking, such as critical analysis and metacognitive skills, remains a significant barrier to its use as a standalone assessment and feedback tool, especially in complex academic writing, which requires less‐predictable outputs [16, 31]. This limitation appears to be inherent to the current programming of LLMs rather than the result of poor prompt engineering, as even iterative refinement failed to elicit deeper critical insight. This core finding, that the output quality relies on the quality of the prompt, echoes research highlighting the ways prompt engineering can yield optimal outputs from GenAI [28, 32, 33].

Despite these limitations, the rapid nature of GenAI feedback offers a potential solution to the limited time available to academic staff in HE for providing feedback and guidance to students. Typically, feedback is provided *post hoc* for assessments, whereas there is a strong argument that it is more impactful when provided *during* the assessment activity, allowing students to modify their behaviours before the summative point of that assessment [34]. This focus on mid‐assignment formative feedback aligns with the observation by Voelkel et al. [14] that students valued feedback provided on the current assignment more than *post hoc* feedback relating to future assignments. By providing viable, actionable feedback, GenAI enables this mid‐assignment formative guidance that would require substantive workload, and a consequential time lag, if it were provided by an academic tutor. The rapidity of GenAI responses also may empower multiple sequential, iterative, real‐time formative interactions with the feedback, at a scale that human educators struggle to match. Furthermore, if GenAI can be reliably used to triage structural and linguistic issues, academics could focus their attention toward those aspects not addressed by GenAI—analysis, criticality, creativity and forward thinking.

GenAI platforms may, therefore, have a role to play in providing formative feedback, but this use must be accompanied by strong caveats that the guidance may contain limitations, omissions and possibly inaccuracies. A specific concern was that while GenAI‐generated feedback might correct misunderstandings in included content, it often fails to identify critical omissions or overlooks key contexts. The feedback was not always comprehensive, and although not entirely inaccurate, it was often limited in coverage and scope, while the focus on structure, language and use of references is potentially insufficient to support a student in gaining good marks. This finding aligns with a recent study which identified that roughly 50% of students in the study used GenAI for feedback on their work [35]. However, both students and educators noted that there were limitations to the feedback, and educator‐provided feedback was trusted more by the students.

As with all GenAI tools, the potential value of the output depends heavily on the quality of the prompt. This study involved an iterative process of prompt refinement, informed by good feedback practice. A student would probably not have the background knowledge of academic writing needed to write an effective prompt. Consequently, if GenAI is to be used formatively, it may be necessary for academics to provide pre‐engineered prompts for students, focusing on specific aspects. Although this requirement assumes that the educators also possess the requisite GenAI skills themselves.

This study aimed to align the GenAI‐generated feedback with models of effective feedback provision. The present design, using single, static GenAI outputs, cannot itself speak to the dialogic dimension of the feedback process; therefore, the observations presented here are offered as directions that the present findings raise, rather than as conclusions the data can empirically support. Contemporary feedback models prioritise a dialogic dimension, where the feedback process is a conversation rather than a static artefact. This dialogic element is central to the efficacy of effective feedback practice [36]. While the feedback provided in the study was specific and offered feed‐forward guidance, it remains a static product for the students to consume passively. As a result, the GenAI feedback may be useful as a benchmark for progress and an indication of specific elements that need to be addressed, but may have a limited impact in advancing learning or the development of the skill being assessed. Adopting a Socratic tutor interaction style for interacting with GenAI has been suggested to better encourage reflection [37, 38]. A balance must, therefore, be struck between providing detailed directives and encouraging deep, reflective learning.

From a psychological perspective, and, again, as a direction for future rather than present investigation, GenAI feedback may offer an advantage regarding student agency and emotional regulation [23]. Pekrun's ‘Control‐value Theory’ [24, 39] highlights that learners' emotions and motivations are influenced by their perceived level of control over the outcomes of an activity. Engaging with a nonhuman entity may reduce the anxiety associated with the ‘achievement emotions’—the emotions induced by (or associated with) success or failure. Managing their emotions is fundamental in affecting the extent to which learners will want to engage with the feedback process, and apply it to future work. Allowing students to control the source of feedback by prompting a LLM, might provide the sense of agency needed to induce achievement emotions that encourage self‐improvement.

Interestingly, the GenAI feedback in this study showed a notable paucity of motivational comments. Research indicates that learners are more engaged when they receive positive commentary [40, 41, 42], aligning with the findings that perceptions of care and self‐efficacy greatly impact how students engage with feedback [43]. However, an intriguing study by Lipnevich et al. [44] identified that praise may actually have a negative effect on students' motivations to act on feedback. These authors suggested that feedback which inferred the student had performed excellently, appeared to induce an anchoring bias—a subconscious impression to the student that they did not need to engage with feedback to improve their work. So, the neutral or even slightly critical tone of GenAI‐generated feedback may be a benefit, preventing this complacency. There is evidence that individual students display a preference for different ‘personalities’ of a platform, and this may therefore influence their trust of, and engagement with, any feedback provided [45].

There is also the potential that the feedback coming from a nonhuman source could be perceived as less negatively judgemental than feedback coming from a human. Aligning again with Pekrun's control‐value theory, feelings of being judged or negatively appraised may have an impact on a student's desire to engage with the guidance within the feedback process, or even access the feedback at all [23]. Removing the human from the interaction has the potential to obviate this concern.

### Limitations and future directions

This study was a small‐scale pilot activity. Two features of the design impact the strength of the conclusions. Firstly, the outputs of LLMs are known to vary subtly from run to run, even for an identical prompt [46]. Although the consistency of outputs was checked during the prompt development stage, both the pilot and the expanded set are reported as a single output per essay, scored once. Therefore, the quality recorded for any given essay represents a single response drawn from a variable distribution, rather than representing a stable property of the LLM. As the artefact is the single output that a student receives, one generation per essay retains a degree of validity, in that a student typically takes the first output returned. This justifies the student‐facing framing; however, rather than any general claims about GenAI feedback, observations made here are confined to, and reflective of, a single interaction, which is not scaffolded by an interaction with a tutor/educator. Secondly, the expanded data set was scored by a single researcher, so that no inter‐rater agreement could be established for these data. A more robust rubric for evaluating the quality of feedback could be developed, though this would need to be tested against human‐provided feedback as a control.

It should be emphasised that this study evaluated GenAI feedback against established principles of good feedback practice, and not against feedback produced by experienced educators. The conclusions are, therefore, limited to alignment with these principles, rather than extending to feedback quality relative to established academic practice. A direct comparison of GenAI‐generated, human‐generated and hybrid feedback represents an important next step and would provide the control against which a more robust rubric could be validated. Additionally, it would be useful to investigate the extent to which the GenAI‐generated feedback changes over an iterative sequence of feedback being received, acted upon and then a revised manuscript submitted for further feedback (and this cycle repeated several times). Finally, it would be important to compare the quality of the GenAI‐generated feedback from the simplistic approach adopted here (no rubric uploaded, and a single feedback artefact produced per essay) to a more idealised approach, using an uploaded rubric, and multiple replicates of the same chat. Also, feedback on more‐complex material, such as critical analyses, reflective accounts or creative work, may lead to different outcomes than feedback on a short Year 1 bioscience essay. As the complexity and nuance within the essays increases, it is likely that GenAI will reach its limitations sooner, and its utility would therefore drop off substantially.

## Conclusions

This study emphasises the importance of centring any evaluation of GenAI‐generated feedback within the broader discussion of good feedback practice and the understanding of how students engage with the feedback process. Adopting GenAI to provide a feedback output strongly reinforces the role of feedback as a product rather than a process. However, combining multiple feedback sources can improve engagement and motivate students to attempt more complex tasks [10].

The potential of using GenAI for providing formative feedback is limited, but not completely null. However, the degree of understanding of prompting, GenAI usage, hallmarks of effective writing, and good feedback practices may place its use outside the reach of those students who would benefit the most from guidance. Any GenAI use would therefore need to be guided and scaffolded strongly by experienced educators. There is still the absolute requirement for the role of an experienced human educator in this process, and while GenAI may help streamline the feedback process, it cannot, at present, replace it entirely.

## Conflict of interest

The authors declare no conflicts of interest.

## Author contributions

AC undertook the primary analysis and drafted the paper; NF codesigned the overall project and contributed to the manuscript; AS codesigned the overall project; SR was the principal investigator, codesigned the project, contributed to primary analysis and produced the submitted version of the paper.

## Supporting information


**Data S1.** Prompts used for GPT4o. The prompts used to elicit feedback comments from the LLM. Prompts are presented in order of development via iterative testing on two sample essays.
**Table S1.** Rubric for evaluating feedback on essays: Details of the bespoke rubric used to evaluate GenAI generated feedback comments. 6 criteria, based on established features of good practice in the provision of written feedback, were evaluated against 5 levels of quality.
**Fig. S1.** Mark distribution of selected essays compared to total pool of essays. The proportions of essays showing the overall grade from the original markers are compared between the pool of essays available, and the essays selected. The selected essays show a similar profile of marks to the original pool.

## Data Availability

The data that support the findings of this study are available from the corresponding author at rutherfords@cf.ac.uk upon reasonable request.

## References

[1] Abubakar S , Jeilani A and Yusuf M (2025) The role of over‐reliance on AI in the negative consequences of student learning: the moderating effects of ethical concerns and institutional policies. Cogent Educ 12, 2591503.

[2] Farhat Z (2025) Hooked on help: student overreliance on ChatGPT in higher education. J Inf Technol Teach Cases 1–8.

[3] van Niekerk J , Delport PMJ and Sutherland I (2025) Addressing the use of generative AI in academic writing. Comput Educ Artif Intell 8, 100342.

[4] Hubert KF , Awa KN and Zabelina DL (2024) The current state of artificial intelligence generative language models is more creative than humans on divergent thinking tasks. Sci Rep 14, 3440. 38341459 10.1038/s41598-024-53303-w PMC10858891

[5] Latif E , Zhou Y , Guo S , Gao Y , Shi L , Nyaaba M , Bewerdorff A , Yang X and Zhai X (2025) Comparative evaluation of OpenAI O1 and human performance in higher order cognition. Sci Rep 16, 3603. 41453997 10.1038/s41598-025-33629-9 PMC12847924

[6] Wagner G , Prester J , Mousavi R , Lukyanenko R and Paré G (2026) Generative artificial intelligence for literature reviews. J Inf Technol 41, 148–170.

[7] Williams A (2024) Delivering effective student feedback in higher education: an evaluation of the challenges and best practice. Int J Res Educ Sci 10, 473–501.

[8] Nicol D (2010) From monologue to dialogue: improving written feedback processes in mass higher education. Assess Eval High Educ 35, 501–517.

[9] Winstone NE , Mathlin G and Nash RA (2019) Building feedback literacy: students' perceptions of the developing engagement with feedback toolkit. Front Educ 4, 39.

[10] Winstone NE , Nash RA , Rowntree J and Parker M (2016) It'd be useful, but I wouldn't use it’: barriers to university students' feedback seeking and recipience. Stud High Educ 42, 2026–2041.

[11] Sullivan S , Gaydon M , Panton S , Nguyen XH and Richardson J (2025) Perceptions of feedback: the use of self‐reflection to improve student satisfaction. Assess Eval High Educ 51, 301–314.

[12] Carless D (2006) Differing perceptions in the feedback process. Stud High Educ 31, 219–233.

[13] Boud D and Molloy E (2013) Rethinking models of feedback for learning: the challenge of design. Assess Eval High Educ 38, 698–712.

[14] Voelkel S , Varga‐Atkins T and Mello LV (2020) Students tell us what good written feedback looks like. FEBS Open Bio 10, 692–706. 10.1002/2211-5463.12841 PMC7193163 32176832

[15] Pitt E and Winstone N (2022) Enabling and valuing feedback literacies. Assess Eval High Educ 48, 149–157.

[16] Yang M and Carless D (2013) The feedback triangle and the enhancement of dialogic feedback processes. Teach High Educ 18, 285–297.

[17] Musaeus P , Prilop CN , Dikilitaş K , Alterskjær Sundset M , Svensen C , Ershova T , Boud D , Büchert Lindberg A , Eggers Bjælde O , Gray RM Jr *et al*. (2025) Teacher feedback literacy in higher education: navigating enablers and constraints. Assess Eval High Educ 51, 315–331.

[18] Henderson M , Ryan T and Phillips M (2019) The challenges of feedback in higher education. Assess Eval High Educ 44, 1237–1252.

[19] Swingler MV and Nicol D (2026) Do students generate better self‐feedback by comparing their work against assessment criteria or exemplars? Assess Eval High Educ 1–20.

[20] Nicol DJ and Macfarlane‐Dick D (2007) Formative assessment and self‐regulated learning: a model and seven principles of good feedback practice. Stud High Educ 31, 199–218.

[21] Winstone N and Carless D (2019) Designing Effective Feedback Processes in Higher Education: A Learning‐Focused Approach. Routledge, London.

[22] Dweck CS (2013) Self‐Theories: Their Role in Motivation, Personality, and Development. Psychology press, New York.

[23] Grant H and Dweck CS (2003) Clarifying achievement goals and their impact. J Pers Soc Psychol 85, 541–553. 14498789 10.1037/0022-3514.85.3.541

[24] Pekrun R (2006) The control‐value theory of achievement emotions: assumptions, corollaries, and implications for educational research and practice. Educ Psychol Rev 18, 315–341.

[25] Andrade H and Du Y (2005) Student perspectives on rubric‐referenced assessment. Pract Assess Res Eval 10, 3.

[26] Gonsalves C (2023) Democratising assessment rubrics for international students. Assess Eval High Educ 49, 587–600.

[27] Graham AI , Harner C and Marsham S (2021) Can assessment‐specific marking criteria and electronic comment libraries increase student engagement with assessment and feedback? Assess Eval High Educ 47, 1071–1086.

[28] Balabdaoui F , Dittmann‐Domenichini N , Grosse H , Schlienger C and Kortemeyer G (2024) A survey on students' use of AI at a technical university. Discov Educ 3, 51.

[29] Sawalha G , Taj I and Shoufan A (2024) Analyzing student prompts and their effect on ChatGPT's performance. Cogent Educ 11, 2397200.

[30] Kiger ME and Varpio L (2020) Thematic analysis of qualitative data: AMEE guide No. 131. Med Teach 42, 846–854. 32356468 10.1080/0142159X.2020.1755030

[31] Hattie J and Timperley H (2007) The power of feedback. Rev Educ Res 77, 81–112.

[32] Jacobsen LJ and Weber KE (2025) The promises and pitfalls of large language models as feedback providers: a study of prompt engineering and the quality of AI‐driven feedback. AI 6, 35.

[33] Mzwri K and Turcsányi‐Szabo M (2025) The impact of prompt engineering and a generative AI‐driven tool on autonomous learning: a case study. Educ Sci 15, 199.

[34] DiFrancesca D , Nietfeld JL and Cao L (2016) A comparison of high and low achieving students on self‐regulated learning variables. Learn Individ Differ 45, 228–236.

[35] Henderson M , Bearman M , Chung J , Fawns T , Buckingham Shum S , Matthews KE and de Mello Heredia J (2025) Comparing generative AI and teacher feedback: student perceptions of usefulness and trustworthiness. Assess Eval High Educ 51, 863–878.

[36] Carless D (2016) Feedback as dialogue. In Encyclopedia of Educational Philosophy and Theory ( Peters M , ed.), pp. 1–6. Springer, Singapore. doi: 10.1007/978-981-287-532-7_389-1

[37] Nahar N (2025) Exploring student perspectives on AI‐generated feedback using a Socratic method chatbot. *JLD*HE.

[38] Chan JCH and Ho KHM (2026) Correction to ‘using GenAI for Socratic questioning: an approach to higher‐order thinking for nursing education’, nursing open. 13, e70467. 10.1002/nop2.70467 PMC13051991 41928662

[39] Pekrun R , Frenzel AC , Goetz T and Perry RP (2007) The control‐value theory of achievement emotions: an integrative approach emotions in education. In Emotion in Education ( Schutz PA and Pekrun R , eds), Academic Press, Amsterdam.

[40] Harlen W and Deakin Crick R (2010) Testing and motivation for learning. Assess Educ Princ Policy Pract 10, 169–207.

[41] Jones H , Hoppitt L , James H , Prendergast J , Rutherford S , Yeoman K and Young M (2012) Exploring students' initial reactions to the feedback they receive on coursework. Biosci Educ 20, 4–21.

[42] Butler R (2011) Enhancing and undermining intrinsic motivation: the effects of task‐involving and ego‐involving evaluation on interest and performance. Br J Educ Psychol 58, 1–14.

[43] Carless D (2018) Feedback loops and the longer‐term: towards feedback spirals. Assess Eval High Educ 44, 705–714.

[44] Lipnevich AA , Eßer FJ , Park MJ and Winstone N (2023) Anchored in praise? Potential manifestation of the anchoring bias in feedback reception. Assess Educ Princ Policy Pract 30, 4–17.

[45] Henderson M , Bearman M , Chung J , Fawns T , Buckingham Shum S , Matthews KE and de Mello Heredia J (2025) Comparing generative AI and teacher feedback: student perceptions of usefulness and trustworthiness. Assess Eval High Educ 51, 1–16.

[46] Ouyang S , Zhang JM , Harman M and Wang M (2025) An empirical study of the non‐determinism of ChatGPT in code generation. ACM Trans Softw Eng Methodol 34, 1–28.

[47] Touron DR and Hertzog C (2014) Accuracy and speed feedback: global and local effects on strategy use. Exp Aging Res 40, 332–356. 24785594 10.1080/0361073X.2014.897150 PMC4009509

[48] Dawson P , Henderson M , Mahoney P , Phillips M , Ryan T , Boud D and Molloy E (2018) What makes for effective feedback: staff and student perspectives. Assess Eval High Educ 44, 25–36.

[49] Truscott J (2007) The effect of error correction on learners' ability to write accurately. J Second Lang Writ 16, 255–272.

[50] Mandouit L and Hattie J (2023) Revisiting “the power of feedback” from the perspective of the learner. Learn Instr 84, 101718.

