# Supplementary material for: Evaluating GenAI‐produced feedback on undergraduate bioscience essays against good higher education feedback practice
Source: FEBS Open Bio. 2026 Sep 4:10.1002/2211-5463.70339. Online ahead of print. doi: 10.1002/2211-5463.70339 (PMC13543122; doi:10.1002/2211-5463.70339)
Supplement: Supplementary file 1 [file FEB4-9999-0-s001.pdf]

SUPPLEMENTARY INFORMATION

Supplementary Information S1: Prompts used for GPT4o. The prompts used to elicit feedback comments from the LLM. Prompts are presented in order of development via iterative testing on two sample essays.

Prompts (in order of development):

Prompt A:

Give me feedback on this essay

Give me detailed feedback on this essay

Give me precise feedback on this essay

Prompt B:

Provide precise feedback on this university-level biology essay as if you were a university professor.

Prompt C:

I want you to act as an AI writing tutor. I will provide you with a student who needs help improving their writing and your task is to use artificial intelligence tools, such as natural language processing, to give the student feedback on how they can improve their composition. You should also use your rhetorical knowledge and experience about effective writing techniques in order to suggest ways that the student can better express their thoughts and ideas in written form. My first request is "Provide precise feedback on this undergraduate level biology essay. Be candid and objective."

Prompt D:

I want you to act as an AI writing tutor. I will provide you with a student who needs help improving their writing and your task is to use artificial intelligence tools, such as natural language processing, to give the student feedback on how they can improve their composition. You should also use your rhetorical knowledge and experience about effective writing techniques in order to suggest ways that the student can better express their thoughts and ideas in written form. My first request is "Provide precise feedback on this undergraduate level biology essay. Be candid and objective. When needed quote the essay and give examples on improved versions".

Prompt E:

Imagine you are a university biology professor tasked with evaluating a student's essay. Your role is to provide detailed, constructive feedback aimed at improving the student's understanding and writing skills. Assess the essay on multiple dimensions, including the depth of biological concepts presented, clarity of argumentation, scientific accuracy, and writing style. Identify strengths and areas for improvement, suggesting concrete steps the student can take to refine their work. Go beyond the immediate essay – offer insights into how the student can enhance their critical thinking, research skills and scientific communication for future assignments. Be candid but supportive, framing your feedback to encourage growth and inspire academic excellence.

Supplementary Table 1

Rubric for Evaluating Feedback on Essays: Details of the bespoke rubric used to evaluate GenAI generated feedback comments. 6 criteria, based n established features of good practice in the provision of written feedback, were evaluated against 5 levels of quality.

Criteria	Below Expectations (Level 1)	Approaching Expectations (Level 2)	Meets Expectations (Level 3)	Exceeds Expectations (Level 4)	Exemplary (Level 5)
Accuracy of Feedback:	Feedback contains i ncorrect or misleading information about essay concepts or content; e.g. "Your essay does not address the topic," when the essay clearly does.	Feedback is partially accurate but includes some errors or misunderstandings; e.g. "Your thesis is missing," when the thesis exists but is weak or unclear.	Feedback is mostly accurate with minor issues; e.g. "Your essay makes a strong point but could benefit from more evidence in paragraph 2."	Feedback is fully accurate and relevant; e.g. "Your essay successfully addresses the topic, but the argument in paragraph 3 needs stronger evidence from the assigned readings."	Feedback demonstrates a sophisticated understanding of the essay's content, not only addressing accuracy but also identifying deeper opportunities for improvement; e.g. "Your essay provides a thorough response to the topic, but the analysis in paragraph 3 could be strengthened by comparing it to the counterargument in paragraph 5. This would create a more balanced and persuasive discussion."
Focus on content:	Feedback is generic, vague, and does not engage with the specific content of the essay; e.g. feedback might state, "This part is confusing," without identifying t he specific section or issue.	Feedback begins to address content but lacks full clarity or detail, making it difficult for the writer to act on; e.g. "Your examples are weak" but fails to identify which examples.	Feedback is clear organised and specifically references parts of the essay; e.g. "Your introduction sets up the topic well, but the thesis statement at the end could be more specific about the main argument you're making in the body paragraphs."	Feedback addresses the essay's content by analysing structure, logic and arguments in detail. It identifies areas for improvement and explains why they are necessary in context with the essay's goals; e.g. "The second paragraph introduces an example, but it doesn't tie back to your thesis on X. Expanding on how this example supports your main argument would clarify its purpose and strengthen your overall position."	Feedback not only identifies specific strengths and weaknesses but also offers a deeper analysis of how the content contributes to the essay's overall success; e.g. "Your introduction effectively sets the tone, but consider tying the hook more directly to your thesis to create a cohesive flow. In paragraph 4, your evidence is strong, but drawing on a real-world example would provide additional depth and make the argument more relatable."

**Specificity, Clarity, Detail**	Feedback is generic, vague, and does not reference the essay; e.g. "This part is confusing."	Feedback is somewhat specific but lacks full clarity or detailed references; e.g. "Your examples are weak" without identifying w hich examples.	Feedback is clear, organized, and references specific parts of the essay; e.g. "The second paragraph needs better transitions to connect with the thesis."	Feedback is highly specific and detailed, with precise references to the essay; e.g. "In the second paragraph, the transition from X to Y is abrupt. Consider adding a sentence to link these ideas."	Feedback demonstrates exceptional clarity and detail, anticipating potential questions or challenges the writer might face when revising; e.g. "In paragraph 3, your transition from X to Y is unclear because the connection between these ideas is implicit. You could explicitly state how X leads to Y, perhaps by adding a sentence like, 'Because X influences Y in this way, the argument becomes more relevant.' This would help clarify your logic."
**Constructive**	Feedback is overly critical or harsh and offers no constructive advice ; e.g. "This essay is poorly written."	Feedback is mostly critical with limited constructive advice; e.g. "Your grammar needs improvement" without suggesting specific areas.	Feedback balances critique and constructive advice; e.g. "Your thesis is clear, but paragraph 3 would improve by linking back to it."	Feedback is highly constructive, offering detailed and actionable suggestions; e.g. "Your thesis is strong. To improve paragraph 3, connect the example of 'X' more explicitly to the thesis argument."	Feedback is not only constructive but also empowering, offering tailored suggestions that encourage creativity and critical thinking; e.g. "Your thesis is strong and clearly presented. In paragraph 3, you could enrich your argument by comparing the example of 'X' to a real-world scenario or contrasting it with a counterexample. This would deepen your analysis and make your essay more impactful."
**Feed forward and Critical**	Suggestions are impractical or vague; e.g. "Do better research."	Suggestions are somewhat actionable but lack clarity or detail; e.g. "Add more evidence," without indicating wh ere or what kind.	Suggestions are practical and mostly clear; e.g. "Add evidence to paragraph 2 to strengthen your argument about X."	Suggestions are highly practical, actionable, and forward-thinking; e.g. "Consider using data from the figure in paragraph 2 to support your argument about X."	Suggestions go beyond immediate revisions to encourage long-term growth and skills development; e.g. "In paragraph 2, adding evidence from the assigned readings will strengthen your argument about X. Going forward, you might consider exploring additional scholarly sources on Y to provide even more nuanced support for your thesis in future essays."

**Motivational**	Tone is negative or discouraging; e.g. “You clearly didn’t try hard enough.”	Tone is neutral or mixed, with minimal encouragement; e.g. “Your argument is okay but not great.”	Tone is positive and encouraging, motivating improvement; e.g. “You’ve made good points here, and with a stronger conclusion, this essay will be excellent.”	Tone is highly encouraging and inspiring; e.g. “You’ve laid a great foundation for your argument! Strengthening the conclusion will make this essay outstanding.”	Feedback not only encourages improvement but also inspires confidence and a sense of pride in the student’s work; e.g. “Your essay shows clear evidence of your hard work and thoughtful analysis. With a few targeted revisions, like refining your conclusion to emphasize the broader implications of your argument, this essay could be truly exceptional. You’re well on your way to mastering scientific writing!”

Supplementary Figure 1

Mark distribution of selected essays compared to total pool of essays. The proportions of essays showing the overall grade from the original markers are compared between the pool of essays available, and the essays selected. The selected essays show a similar profile of marks to the original pool.

Figure S1

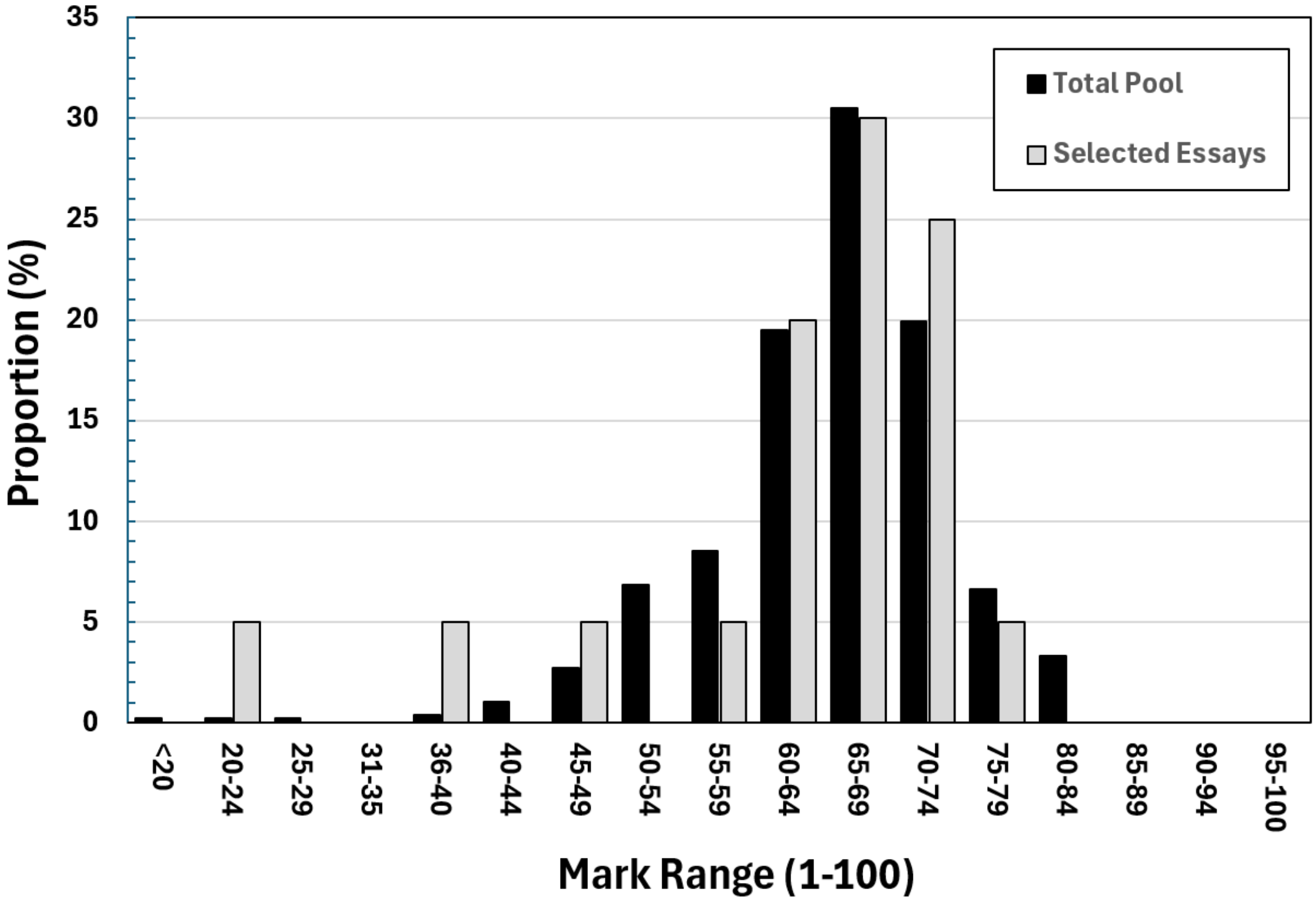

Mark distribution of selected essays compared to overall pool. The distribution of the overall mark for the essays selected for analysis (grey bars, n = 30) compared to the total essay pool (black bars, n = 460). Data bins represent 5-point ranges on a scale of 1-100.
